# (*E*)-3-[1-(2,4-Difluoro­phen­yl)eth­yl]-5-methyl-*N*-nitro-1,3,5-oxadiazinan-4-imine

**DOI:** 10.1107/S1600536810026425

**Published:** 2010-07-10

**Authors:** Yuan-yuan Zhong, Cong-cong Li, Liang-zhong Xu

**Affiliations:** a College of Chemistry and Molecular Engineering, Qingdao University of Science and Technology, Qingdao 266042, People’s Republic of China

## Abstract

The 1,3,5-oxadiazinane ring in the title compound, C_12_H_14_F_2_N_4_O_3_, has a conformation inter­mediate between half-chair and screw-boat. The crystal structure is stabilized by weak inter­molecular C—H⋯O hydrogen bonds. Weak π–π inter­actions are indicated by the relatively long centroid–centroid distance of 3.9199 (12) Å and inter­planar distance of 3.803 Å between symmetry-related benzene rings from neighbouring mol­ecules.

## Related literature

An important type of insecticide, oxadiazine compounds are highly efficient and of low toxicity, see: Gsell *et al.*(1998 ▶). The title compound has been used to synthesize many similar insecticides, see: Maienfisch *et al.* (1994 ▶). For the preparation of the title compound, see: Gottfied *et al.*(2001 ▶). For the related structures, see: Chopra *et al.*, (2004 ▶); Kang *et al.* (2008 ▶). For puckering parameters, see: Cremer & Pople (1975 ▶). 
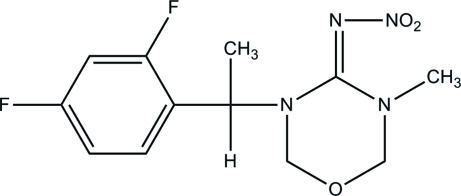


## Experimental

### 

#### Crystal data

C_12_H_14_F_2_N_4_O_3_
*M* *_r_* = 300.27
Monoclinic, 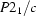

*a* = 13.385 (3) Å
*b* = 6.7470 (13) Å
*c* = 15.073 (3) Å
β = 101.25 (3)°
*V* = 1335.0 (5) Å^3^
*Z* = 4
Cu *K*α radiation
μ = 1.11 mm^−1^
*T* = 113 K
0.26 × 0.24 × 0.22 mm

#### Data collection

Rigaku Saturn diffractometer
Absorption correction: numerical (*CrystalClear*; Rigaku, 2005 ▶) *T*_min_ = 0.762, *T*_max_ = 0.793
13266 measured reflections
2567 independent reflections
2168 reflections with *I* > 2σ(*I*)
*R*_int_ = 0.061

#### Refinement

*R*[*F* ^2^ > 2σ(*F* ^2^)] = 0.040
*wR*(*F* ^2^) = 0.106
*S* = 1.09
2567 reflections
193 parameters
H-atom parameters constrained
Δρ_max_ = 0.31 e Å^−3^
Δρ_min_ = −0.30 e Å^−3^

### 

Data collection: *CrystalClear* (Rigaku, 2005 ▶); cell refinement: *CrystalClear*; data reduction: *CrystalClear*; program(s) used to solve structure: *SHELXS97* (Sheldrick, 2008 ▶); program(s) used to refine structure: *SHELXL97* (Sheldrick, 2008 ▶); molecular graphics: *ORTEPIII* (Burnett & Johnson, 1996 ▶) and *ORTEP-3 for Windows* (Farrugia, 1997 ▶); software used to prepare material for publication: *SHELXTL* (Sheldrick, 2008 ▶).

## Supplementary Material

Crystal structure: contains datablocks I, global. DOI: 10.1107/S1600536810026425/dn2586sup1.cif

Structure factors: contains datablocks I. DOI: 10.1107/S1600536810026425/dn2586Isup2.hkl

Additional supplementary materials:  crystallographic information; 3D view; checkCIF report

## Figures and Tables

**Table 1:** Hydrogen-bond geometry (Å, °)

*D*—H⋯*A*	*D*—H	H⋯*A*	*D*⋯*A*	*D*—H⋯*A*
C1—H1*A*⋯O3^i^	0.99	2.50	3.1908 (16)	127
C3—H3*A*⋯O2^ii^	0.99	2.51	3.4439 (18)	156
C4—H4*C*⋯O2^iii^	0.98	2.49	3.1665 (17)	126
C6—H6*A*⋯O3^iv^	0.98	2.39	3.2046 (18)	140

Symmetry codes: (i) 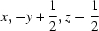; (ii) 
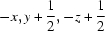
; (iii) 
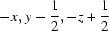
; (iv) 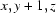.

**Fig. 1.**
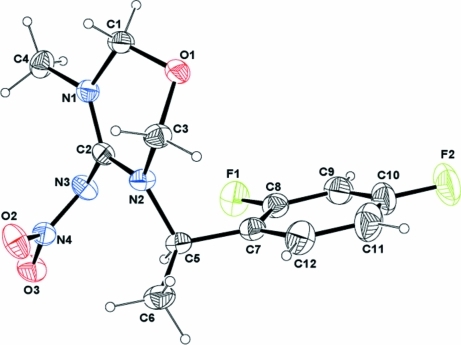
View of the title compound (I), with the atom labeling scheme. Displacement ellipsoids are drawn at the 50% probability level.H atoms are represented as small spheres of arbitrary radii.

## supplementary crystallographic information

### Comment

As an important type of insecticides, oxadiazine compounds are highly efficient and of low toxicity (Gsell, *et al.*, 1998). Lots of similar insecticides compounds were synthesized with the title compounds (I) (Maienfisch, *et al.*, 1994). We report the synthesis and crystal structure of the title compound, (I).

The conformation of the 1,3,5-oxadiazinane ring in(I)is intermediate between half-chair and screw-boat with puckering parameters (Cremer & Pople, 1975): Q= 0.5303 (12)Å; θ= 59.14 (13)°; φ= 329,54 (15)°. The benzene ring forms dihedral angles of 74.84 (3)° and 87.30 (2)° with the mean plane of the oxadiazine ring. The bond lengths and angles of the oxadiazine rings are in a good agreement with those reported previously (Chopra, *et al.*, 2004). The N=C bond length [N3=C2 = 1.3804 (2) Å] are close to the value reported in the literature (Kang,*et al.*,2008).

The structure is stabilized by hydrogen bonds of C-H···O type. And with a π-π stacking between symmetry related phenyl rings with a centroid-to-centroid distance of 3.9199 (12)Å and interplanar distance of 3.803Å resulting in a 0.951Å slippage.

### Experimental

1-(1-bromoethyl)-2,4-difluorobenzene 4.5 g (20.0 mmol),(*Z*)-3-methyl-N– nitro-1,3,5-oxadiazinan-4-imine 3.2 g (20.0 mmol), potassium carbonate 2.8 g (20.0 mmol) and acetonitril 20 g were charged in a flask equipped with stirrer, water separator and reflux condenser. The mixture was heated to reflux for 4 h. Upon cooling at room temperature. The reaction mixture was filtered, and the solution was concentrated under reduced pressure to give the title compound (I) 4.5 g (76% yield). (Gottfied, *et al.*, 2001). Single crystals suitable for X-ray measurement were grown by slow evaporation of an ethanol solution of (I).

### Refinement

All H atoms were fixed geometrically and treated as riding with C—H = 0.95Å (aromatic), 0.98 Å (methyl), 0.99 Å (methylene) and 1.0 Å (methine) with U_iso_(H) = 1.2U_eq_(C) or U_iso_(H) = 1.5U_eq_(methyl).

### Crystal data

**Table:**

C_12_H_14_F_2_N_4_O_3_	*F*(000) = 624
*M**_r_* = 300.27	*D*_x_ = 1.494 Mg m^−^^3^
Monoclinic, *P*2_1_/*c*	Cu *K*α radiation, λ = 1.54187 Å
Hall symbol: -P 2ybc	Cell parameters from 1502 reflections
*a* = 13.385 (3) Å	θ = 27.7–72.0°
*b* = 6.7470 (13) Å	µ = 1.11 mm^−^^1^
*c* = 15.073 (3) Å	*T* = 113 K
β = 101.25 (3)°	Prism, colorless
*V* = 1335.0 (5) Å^3^	0.26 × 0.24 × 0.22 mm
*Z* = 4	

### Data collection

**Table:**

Rigaku Saturn diffractometer	2567 independent reflections
Radiation source: fine-focus sealed tube	2168 reflections with *I* > 2σ(*I*)
graphite	*R*_int_ = 0.061
Detector resolution: 14.63 pixels mm^-1^	θ_max_ = 72.3°, θ_min_ = 3.4°
ω scans	*h* = −16→15
Absorption correction: numerical (*CrystalClear*; Rigaku, 2005)	*k* = −7→7
*T*_min_ = 0.762, *T*_max_ = 0.793	*l* = −17→18
13266 measured reflections	

### Refinement

**Table:**

Refinement on *F*^2^	Secondary atom site location: difference Fourier map
Least-squares matrix: full	Hydrogen site location: inferred from neighbouring sites
*R*[*F*^2^ > 2σ(*F*^2^)] = 0.040	H-atom parameters constrained
*wR*(*F*^2^) = 0.106	*w* = 1/[σ^2^(*F*_o_^2^) + (0.0692*P*)^2^ + 0.0616*P*] where *P* = (*F*_o_^2^ + 2*F*_c_^2^)/3
*S* = 1.09	(Δ/σ)_max_ < 0.001
2567 reflections	Δρ_max_ = 0.31 e Å^−^^3^
193 parameters	Δρ_min_ = −0.30 e Å^−^^3^
0 restraints	Extinction correction: *SHELXL97* (Sheldrick, 2008), Fc^*^=kFc[1+0.001xFc^2^λ^3^/sin(2θ)]^-1/4^
Primary atom site location: structure-invariant direct methods	Extinction coefficient: 0.0131 (11)

### Special details

**Table:**

Geometry. All e.s.d.'s (except the e.s.d. in the dihedral angle between two l.s. planes) are estimated using the full covariance matrix. The cell e.s.d.'s are taken into account individually in the estimation of e.s.d.'s in distances, angles and torsion angles; correlations between e.s.d.'s in cell parameters are only used when they are defined by crystal symmetry. An approximate (isotropic) treatment of cell e.s.d.'s is used for estimating e.s.d.'s involving l.s. planes.
Refinement. Refinement of *F*^2^ against ALL reflections. The weighted *R*-factor *wR* and goodness of fit *S* are based on *F*^2^, conventional *R*-factors *R* are based on *F*, with *F* set to zero for negative *F*^2^. The threshold expression of *F*^2^ > σ(*F*^2^) is used only for calculating *R*-factors(gt) *etc*. and is not relevant to the choice of reflections for refinement. *R*-factors based on *F*^2^ are statistically about twice as large as those based on *F*, and *R*- factors based on ALL data will be even larger.

### Fractional atomic coordinates and isotropic or equivalent isotropic displacement parameters (Å^2^)

**Table:**

	*x*	*y*	*z*	*U*_iso_*/*U*_eq_	
F1	0.40932 (6)	0.24125 (13)	0.33990 (6)	0.0436 (3)	
F2	0.67967 (7)	0.68795 (18)	0.36250 (8)	0.0676 (4)	
O1	0.23598 (6)	0.67016 (13)	0.16461 (6)	0.0279 (2)	
O2	0.04094 (7)	0.32758 (14)	0.35714 (7)	0.0374 (3)	
O3	0.09896 (7)	0.03447 (15)	0.39933 (6)	0.0365 (3)	
N1	0.13558 (7)	0.38098 (16)	0.16500 (6)	0.0230 (2)	
N2	0.21022 (7)	0.52761 (15)	0.29879 (6)	0.0235 (2)	
N3	0.16278 (8)	0.18616 (15)	0.29436 (7)	0.0272 (3)	
N4	0.09994 (7)	0.18567 (15)	0.35200 (7)	0.0246 (3)	
C1	0.16316 (9)	0.5470 (2)	0.11134 (8)	0.0274 (3)	
H1A	0.1910	0.4946	0.0598	0.033*	
H1B	0.1015	0.6254	0.0866	0.033*	
C2	0.16748 (8)	0.36822 (18)	0.25358 (8)	0.0220 (3)	
C3	0.20435 (10)	0.71093 (19)	0.24732 (8)	0.0261 (3)	
H3A	0.1336	0.7618	0.2352	0.031*	
H3B	0.2492	0.8126	0.2820	0.031*	
C4	0.07765 (10)	0.2205 (2)	0.11312 (9)	0.0302 (3)	
H4A	0.1237	0.1110	0.1065	0.045*	
H4B	0.0455	0.2697	0.0532	0.045*	
H4C	0.0250	0.1736	0.1450	0.045*	
C5	0.27466 (9)	0.51446 (19)	0.39058 (7)	0.0240 (3)	
H5	0.2741	0.3734	0.4108	0.029*	
C6	0.22926 (10)	0.6404 (2)	0.45658 (8)	0.0330 (3)	
H6A	0.2235	0.7781	0.4355	0.050*	
H6B	0.2735	0.6344	0.5165	0.050*	
H6C	0.1615	0.5897	0.4602	0.050*	
C7	0.38383 (9)	0.5676 (2)	0.38557 (8)	0.0265 (3)	
C8	0.44655 (10)	0.4260 (2)	0.35833 (8)	0.0299 (3)	
C9	0.54602 (10)	0.4619 (3)	0.34965 (9)	0.0401 (4)	
H9	0.5870	0.3615	0.3307	0.048*	
C10	0.58224 (10)	0.6488 (3)	0.36972 (11)	0.0442 (4)	
C11	0.52547 (12)	0.7976 (3)	0.39754 (12)	0.0480 (4)	
H11	0.5533	0.9261	0.4112	0.058*	
C12	0.42598 (11)	0.7545 (2)	0.40513 (10)	0.0380 (3)	
H12	0.3856	0.8557	0.4242	0.046*	

### Atomic displacement parameters (Å^2^)

**Table:**

	*U*^11^	*U*^22^	*U*^33^	*U*^12^	*U*^13^	*U*^23^
F1	0.0385 (5)	0.0347 (5)	0.0584 (6)	0.0058 (4)	0.0117 (4)	−0.0135 (4)
F2	0.0272 (5)	0.0938 (9)	0.0812 (8)	−0.0096 (5)	0.0090 (4)	0.0320 (6)
O1	0.0302 (5)	0.0302 (5)	0.0240 (4)	−0.0087 (4)	0.0076 (3)	0.0012 (3)
O2	0.0327 (5)	0.0287 (6)	0.0561 (6)	0.0059 (4)	0.0219 (4)	0.0054 (4)
O3	0.0432 (6)	0.0312 (6)	0.0361 (5)	−0.0011 (4)	0.0104 (4)	0.0155 (4)
N1	0.0241 (5)	0.0235 (6)	0.0218 (5)	−0.0033 (4)	0.0051 (4)	−0.0011 (4)
N2	0.0298 (5)	0.0210 (6)	0.0193 (5)	−0.0006 (4)	0.0041 (4)	0.0004 (4)
N3	0.0332 (6)	0.0203 (6)	0.0312 (6)	0.0007 (4)	0.0139 (4)	0.0022 (4)
N4	0.0255 (5)	0.0232 (6)	0.0249 (5)	−0.0001 (4)	0.0043 (4)	0.0041 (4)
C1	0.0324 (6)	0.0294 (7)	0.0201 (6)	−0.0057 (5)	0.0048 (4)	0.0016 (5)
C2	0.0220 (5)	0.0225 (6)	0.0229 (6)	0.0016 (4)	0.0082 (4)	0.0000 (4)
C3	0.0327 (6)	0.0222 (7)	0.0228 (6)	−0.0016 (5)	0.0038 (5)	0.0012 (4)
C4	0.0280 (6)	0.0294 (7)	0.0317 (6)	−0.0035 (5)	0.0022 (5)	−0.0082 (5)
C5	0.0274 (6)	0.0251 (7)	0.0193 (6)	0.0029 (5)	0.0038 (4)	0.0007 (4)
C6	0.0339 (7)	0.0420 (8)	0.0227 (6)	0.0089 (6)	0.0043 (5)	−0.0043 (5)
C7	0.0283 (6)	0.0286 (7)	0.0218 (6)	0.0020 (5)	0.0030 (4)	0.0023 (5)
C8	0.0297 (6)	0.0325 (8)	0.0271 (6)	0.0030 (5)	0.0041 (5)	0.0006 (5)
C9	0.0291 (7)	0.0560 (10)	0.0356 (7)	0.0096 (7)	0.0074 (5)	0.0064 (7)
C10	0.0239 (7)	0.0612 (11)	0.0458 (8)	−0.0045 (7)	0.0026 (6)	0.0193 (7)
C11	0.0405 (8)	0.0409 (10)	0.0587 (10)	−0.0124 (7)	−0.0004 (7)	0.0099 (7)
C12	0.0372 (7)	0.0305 (8)	0.0447 (8)	−0.0009 (6)	0.0039 (6)	−0.0008 (6)

### Geometric parameters (Å, °)

**Table:**

F1—C8	1.3508 (17)	C4—H4A	0.9800
F2—C10	1.3555 (16)	C4—H4B	0.9800
O1—C1	1.4071 (15)	C4—H4C	0.9800
O1—C3	1.4195 (15)	C5—C7	1.5207 (16)
O2—N4	1.2531 (14)	C5—C6	1.5220 (16)
O3—N4	1.2463 (13)	C5—H5	1.0000
N1—C2	1.3229 (15)	C6—H6A	0.9800
N1—C4	1.4656 (16)	C6—H6B	0.9800
N1—C1	1.4703 (15)	C6—H6C	0.9800
N2—C2	1.3402 (16)	C7—C8	1.3859 (18)
N2—C3	1.4540 (16)	C7—C12	1.389 (2)
N2—C5	1.4834 (15)	C8—C9	1.3843 (18)
N3—N4	1.3219 (14)	C9—C10	1.364 (2)
N3—C2	1.3804 (16)	C9—H9	0.9500
C1—H1A	0.9900	C10—C11	1.373 (3)
C1—H1B	0.9900	C11—C12	1.389 (2)
C3—H3A	0.9900	C11—H11	0.9500
C3—H3B	0.9900	C12—H12	0.9500
			
C1—O1—C3	108.88 (9)	H4B—C4—H4C	109.5
C2—N1—C4	121.56 (10)	N2—C5—C7	109.21 (9)
C2—N1—C1	122.59 (10)	N2—C5—C6	110.04 (10)
C4—N1—C1	115.64 (10)	C7—C5—C6	114.27 (11)
C2—N2—C3	115.97 (10)	N2—C5—H5	107.7
C2—N2—C5	122.63 (10)	C7—C5—H5	107.7
C3—N2—C5	120.64 (10)	C6—C5—H5	107.7
N4—N3—C2	112.64 (10)	C5—C6—H6A	109.5
O3—N4—O2	120.86 (10)	C5—C6—H6B	109.5
O3—N4—N3	117.21 (10)	H6A—C6—H6B	109.5
O2—N4—N3	121.88 (10)	C5—C6—H6C	109.5
O1—C1—N1	110.87 (9)	H6A—C6—H6C	109.5
O1—C1—H1A	109.5	H6B—C6—H6C	109.5
N1—C1—H1A	109.5	C8—C7—C12	116.37 (12)
O1—C1—H1B	109.5	C8—C7—C5	119.71 (12)
N1—C1—H1B	109.5	C12—C7—C5	123.90 (12)
H1A—C1—H1B	108.1	F1—C8—C9	117.73 (12)
N1—C2—N2	118.86 (11)	F1—C8—C7	118.46 (11)
N1—C2—N3	118.27 (11)	C9—C8—C7	123.80 (14)
N2—C2—N3	122.66 (11)	C10—C9—C8	116.62 (14)
O1—C3—N2	108.03 (10)	C10—C9—H9	121.7
O1—C3—H3A	110.1	C8—C9—H9	121.7
N2—C3—H3A	110.1	F2—C10—C9	117.92 (15)
O1—C3—H3B	110.1	F2—C10—C11	118.73 (15)
N2—C3—H3B	110.1	C9—C10—C11	123.35 (13)
H3A—C3—H3B	108.4	C10—C11—C12	117.93 (15)
N1—C4—H4A	109.5	C10—C11—H11	121.0
N1—C4—H4B	109.5	C12—C11—H11	121.0
H4A—C4—H4B	109.5	C7—C12—C11	121.92 (15)
N1—C4—H4C	109.5	C7—C12—H12	119.0
H4A—C4—H4C	109.5	C11—C12—H12	119.0
			
C2—N3—N4—O3	−172.41 (10)	C2—N2—C5—C6	−121.23 (12)
C2—N3—N4—O2	10.03 (16)	C3—N2—C5—C6	69.16 (14)
C3—O1—C1—N1	−47.20 (13)	N2—C5—C7—C8	−81.41 (14)
C2—N1—C1—O1	7.42 (16)	C6—C5—C7—C8	154.88 (11)
C4—N1—C1—O1	−167.37 (10)	N2—C5—C7—C12	97.20 (13)
C4—N1—C2—N2	−172.76 (10)	C6—C5—C7—C12	−26.51 (17)
C1—N1—C2—N2	12.76 (16)	C12—C7—C8—F1	179.02 (11)
C4—N1—C2—N3	12.45 (16)	C5—C7—C8—F1	−2.26 (17)
C1—N1—C2—N3	−162.04 (10)	C12—C7—C8—C9	−0.32 (19)
C3—N2—C2—N1	8.56 (15)	C5—C7—C8—C9	178.39 (11)
C5—N2—C2—N1	−161.50 (10)	F1—C8—C9—C10	−179.21 (12)
C3—N2—C2—N3	−176.88 (10)	C7—C8—C9—C10	0.1 (2)
C5—N2—C2—N3	13.05 (16)	C8—C9—C10—F2	179.50 (12)
N4—N3—C2—N1	−116.27 (12)	C8—C9—C10—C11	0.2 (2)
N4—N3—C2—N2	69.15 (14)	F2—C10—C11—C12	−179.59 (13)
C1—O1—C3—N2	67.89 (12)	C9—C10—C11—C12	−0.2 (2)
C2—N2—C3—O1	−48.47 (13)	C8—C7—C12—C11	0.2 (2)
C5—N2—C3—O1	121.80 (11)	C5—C7—C12—C11	−178.43 (13)
C2—N2—C5—C7	112.59 (12)	C10—C11—C12—C7	0.0 (2)
C3—N2—C5—C7	−57.02 (14)		

### Hydrogen-bond geometry (Å, °)

**Table:**

*D*—H···*A*	*D*—H	H···*A*	*D*···*A*	*D*—H···*A*
C1—H1A···O3^i^	0.99	2.50	3.1908 (16)	127
C3—H3A···O2^ii^	0.99	2.51	3.4439 (18)	156
C4—H4C···O2^iii^	0.98	2.49	3.1665 (17)	126
C6—H6A···O3^iv^	0.98	2.39	3.2046 (18)	140

Symmetry codes: (i) *x*, −*y*+1/2, *z*−1/2; (ii) −*x*, *y*+1/2, −*z*+1/2; (iii) −*x*, *y*−1/2, −*z*+1/2; (iv) *x*, *y*+1, *z*.
